# Compartmental analysis of three-dimensional choroidal vascularity and thickness of myopic eyes in young adults using SS-OCTA

**DOI:** 10.3389/fphys.2022.916323

**Published:** 2022-09-07

**Authors:** Huan Luo, Jinfu Sun, Lan Chen, Dandan Ke, Zheng Zhong, Xi Cheng, Huimin Yu, Xufang Sun

**Affiliations:** ^1^ Department of Ophthalmology, Tongji Hospital, Tongji Medical College, Huazhong University of Science and Technology, Wuhan, Hubei, China

**Keywords:** swept-source optical coherence tomography angiography (SS-OCTA), three-dimensional choroidal vessel volume, choroidal vessel index, axial length (AL), myopia

## Abstract

**Purpose:** We aimed to investigate the change of three-dimensional (3D) choroidal thickness (ChT), choroidal vessel volume (CVV), and choroidal vessel index (CVI) in young myopic adults using swept-source optical coherence tomography angiography (SS-OCTA) and compare the difference of these indicators in different quadrants of the macula and optic disc.

**Methods:** A total of 248 eye samples from 135 participants were used in this cross-sectional study. Each participant underwent detailed history taking and ocular examinations. Based on axial length (AL), patients were divided into the emmetropia (EM) group, mild-myopia (MIM) group, moderate-myopia (MOM) group, and high-myopia (HM) group. 6 mm × 6 mm (1,024 × 1024 B-scans) SS-OCTA scans were performed centered on the fovea and optic disc. 3D ChT, CVV, and CVI were measured based on a built-in deep learning algorithm. Differences in ChT, CVV, and CVI were analyzed in different regions and different myopic groups.

**Results:** Significant reduction in the global CVV were found in the HM group (1.930 ± 0.865) in comparison with the EM (3.486 ± 0.992), MIM (3.238 ± 1.033), and MOM (2.589 ± 1.083) groups (*p* < 0.001). The global CVI was also lower in the HM group (0.258 ± 0.061) than in the EM (0.320 ± 0.055), MIM (0.320 ± 0.051), and MOM (0.286 ± 0.066) groups (*p* < 0.001). The ChT was thinner in eyes with HM (242.753 ± 65.641) than in eyes with EM (377.532 ± 80.593), MIM (348.367 ± 78.191), or MOM (300.197 ± 87.175) (*p* < 0.001). Compartmental analysis revealed that ChT, CVV, and CVI in the nasal quadrant of the macula and temporal and inferior quadrants of the optic disc were much lower than those in other quadrants (*p* < 0.05). Correlation analyses found that ChT, CVV, and CVI were negatively correlated with AL and spherical equivalence.

**Conclusion:** 3D ChT, CVV, and CVI gradually decreased as the degree of myopia increased. The changes were more dramatic on the nasal side of the macula and the temporal and inferior sides of the optic disc. These findings demonstrated the 3D choroidal change and highlighted the papillo-macular bundle as a sensitive region in myopic development.

## Introduction

Myopia is a major public health problem and a leading cause of preventable blindness in children and young adults (Morgan et al., 2012). Multiple epidemiological studies have shown that the prevalence of myopia is increasing globally, especially in Asian countries (Holden et al., 2016; Morgan et al., 2018; Baird et al., 2020).

Optical coherence tomography angiography (OCTA) is an important breakthrough technology in fundus imaging, and its high resolution makes it the only technique suitable for quantifying fundus blood perfusion (Kashani et al., 2017). Alterations in retinal circulation were largely researched in myopia using spectral-domain optical coherence tomography angiography (SD-OCTA) (Al-Sheikh et al., 2017; Li et al., 2018). With the increase of axial length (AL), vessel density (VD) in the macular region and radial peripapillary capillary (RPC) density in the papillary region gradually reduced. However, little attention has been given to changes in choroidal blood perfusion.

The development of swept-source OCTA (SS-OCTA), with faster scan speed, longer wavelength, and deeper penetration, makes it possible to quantify choroidal perfusion change (Siggel et al., 2022; Vagge et al., 2022; Wang et al., 2022). Very recently, choroidal vessel volume (CVV) and choroidal vessel index (CVI) were used to illustrate choroidal blood perfusion in fundus diseases, such as central serous chorioretinopathy (Hwang et al., 2022), age-related macular degeneration (Corbelli et al., 2022), and diabetes retinopathy (Nicolini et al., 2022). CVV is defined as the volume of the large and medium choroidal vessels, which is also known as choroidal luminal volume (LV). CVI is calculated as the ratio of the CVV to the volume of the entire choroid (Singh et al., 2019; Agrawal et al., 2020). However, the binarization algorithm, which was widely used in previous research for calculating CVV and CVI, was only used in B-scan images crossing through the fovea and was limited in illustrating volumetric details.

The aim of this study was to apply a three-dimensional (3D) quantification method to detect choroidal blood perfusion change in young myopic adults. In addition, we aimed to provide a more detailed understanding of the topographic distributions of choroidal alteration by compartmental analysis of both the macula and optic disc.

## Methods

### Study participants

This observational cross-sectional study was approved by the ethics committee of Tongji Hospital, Tongji Medical College, Huazhong University of Science and Technology, Wuhan, China, and was conducted from June 2021 to September 2021 by following the ethical standards stated in the Declaration of Helsinki. Written informed consent was obtained from all the subjects.

A total of 135 subjects between 18 and 30 years of age were enrolled at the ophthalmology clinic of Tongji Hospital. The exclusion criteria were as follows: 1) abnormal intraocular pressure (IOP); 2) evidence or history of ocular diseases, including glaucoma, cataract, and macular edema; 3) evidence or history of systemic disorders, including diabetes, systemic hypertension, and other disorders that can affect the choroid; 4) history of previous ocular surgery; 5) eyes diagnosed with myopic maculopathy according to the ATN classification and grading system (Ruiz-Medrano et al., 2019); 6) subjects who withdrew from the test; and 7) an image signal intensity less than 8 or presence of substantial motion artifacts or center shifts. All the participants underwent comprehensive ophthalmic examinations, including refractive error assessment (AR-310A, Nidek, Japan), IOP measurement (NT-510, Nidek, Japan), AL examination (AL-Scan, Nidek, Japan), color fundus photography (AFC-210, Nidek, Japan), and scanning laser ophthalmoscopy (SLO) (Daytona (PT200), Optos, United Kingdom). Refraction error was converted to spherical equivalent (SE), which was calculated as the spherical diopter with half of the cylindrical diopter (Li et al., 2017).

The subjects were divided into four groups, referred to previous studies (Yang et al., 2017; Khan et al., 2020; Liu et al., 2021): the emmetropia (EM) group, defined by AL<24 mm; the mild-myopia (MIM) group, AL ≥ 24 mm but <25 mm; the moderate-myopia (MOM) group, AL ≥ 25 mm but <26 mm; and the high-myopia (HM) group, AL ≥ 26 mm.

### Image acquisition

An SS-OCTA system (VG200S; SVision Imaging, Henan, China) was used with an SS laser with a central wavelength of approximately 1,050 nm and a scan rate of 200,000 A-scans per second. The resolution was 5 μm in the axial direction and 15 μm in the lateral direction, and the scan depth was 3 mm. Each participant was examined in a dark room between 2 and 5 p.m. and meditated for 10 min before the examination to minimize daily changes in choroidal perfusion and other confounding effects (Lee et al., 2014; Chiang et al., 2015; Woodman-Pieterse et al., 2015). Each eye underwent 6 mm × 6 mm (1,024 × 1024 B-scans) OCTA scans centered on the fovea and optic disc, with each B-scan position scanned four times and averaged (Supplementary Figure S1). To remove eye-motion artifacts, the system includes an eye-tracking tool based on an integrated confocal scanning laser ophthalmoscope. A trained examiner (J.F.S) scanned participants twice in the same region of each eye and then assessed the highest-quality image from the two resulting photos.

### Image processing

First, we used Littmann’s method and Bennett’s formula to adjust the image size according to each AL since a greater AL would result in a larger acquired image owing to view magnification, using previous studies as a reference. The relationship between the actual size (RS) and the recorded image size (CS) can be stated as RS = CS × q2, where q denotes the correction factor, defined as q = 3.382 × 0.013062 × (AL-1.82) according to Bennett’s formula (Bennett et al., 1994; Moghimi et al., 2012; Yang et al., 2016; Li et al., 2017). Each image was corrected in accordance with the correction factor.

**FIGURE 1:**
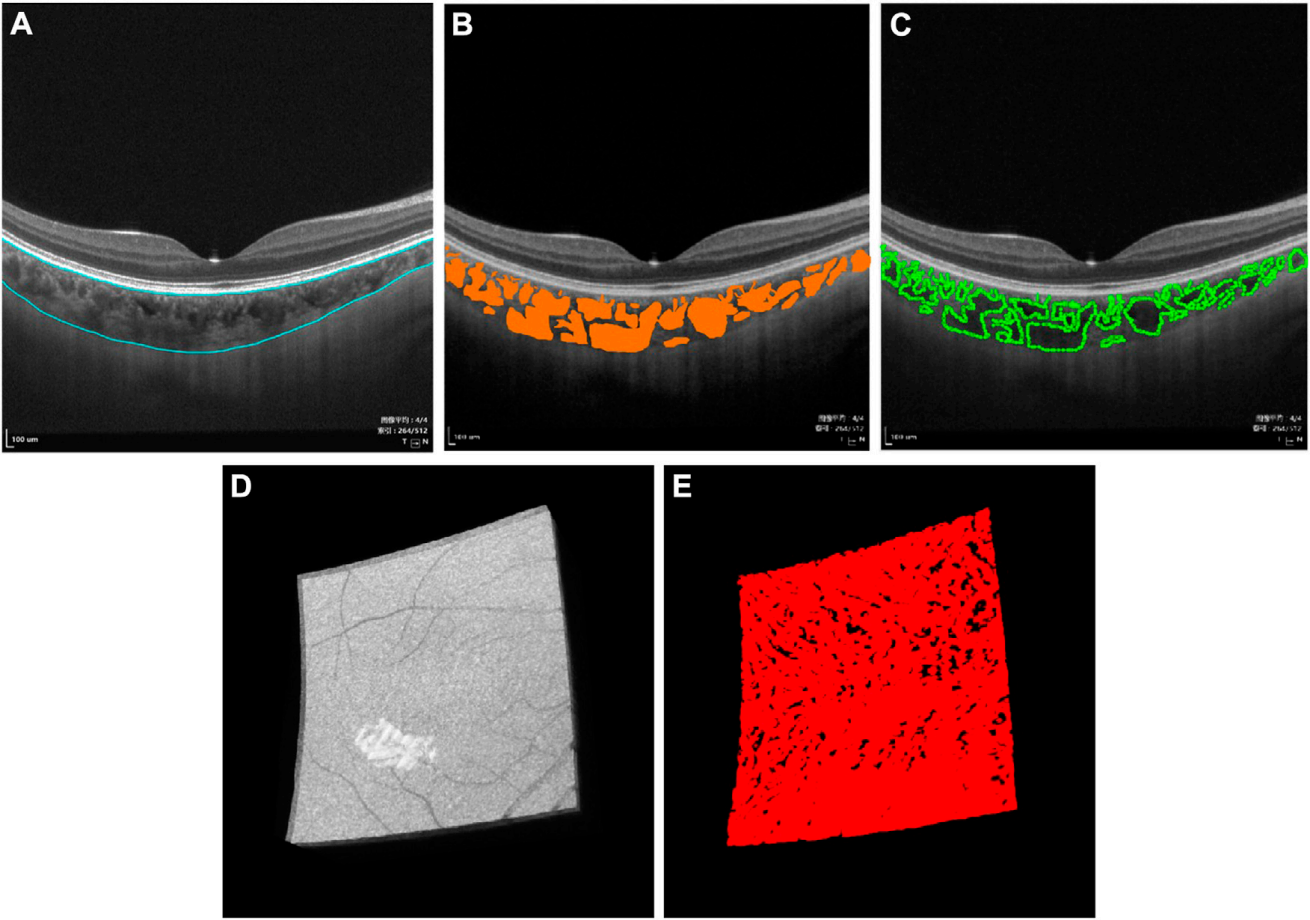
Three-dimensional (3D) representation and quantification of choroidal vascularity index (CVI). **(A)** Raw B-scan image of SS-OCTA with the superior and inferior boundary of the choroid (blue curve). **(B)** Choroidal thickness and choroidal lumen (orange area) were identified by deep learning. **(C)** Choroidal lumen was double-blind manually marked on the obtained B-scan images, which used the LabelMe tool (v3.11.2, https://github.com/wkentaro/labelme) by an experienced ophthalmologist (H.L.), demonstrating excellent coherence with **(B)**. **(D)** Raw 3D choroid image in the macular region of 6 mm × 6 mm acquired by SS-OCTA. **(E)** 3D CVI image generated from **(D)** by deep learning.

Then, segmentation was performed (Figure 1). After automated segmentation using a proprietary built-in algorithm in the SS-OCTA device, a trained examiner (H.L.) checked and manually modified the wrong segmentation.

Thereafter, choroidal thickness (ChT), CVV, and CVI for the entire 3D scanning areas were automatically quantified by a deep learning algorithm in the SS-OCTA device, same as in previous studies (Figure 1, Supplementary Figure S1) (Yang et al., 2020; Sun et al., 2021). ChT was defined as the length between 10 μm above Bruch’s membrane and the choroid–sclera interface. CVV was defined as the volume of the large and medium choroidal vessels. CVI was calculated as the ratio of the CVV to the volume of the entire choroid. In order to verify the accuracy of the CVI value, choroidal luminae were also manually marked in several B-scan images by an experienced ophthalmologist (H.L.) using the LabelMe tool (v3.11.2, https://github.com/wkentaro/labelme). A high degree of agreement was observed between the deep learning algorithm and the manual mark and calculation.

**FIGURE 2:**
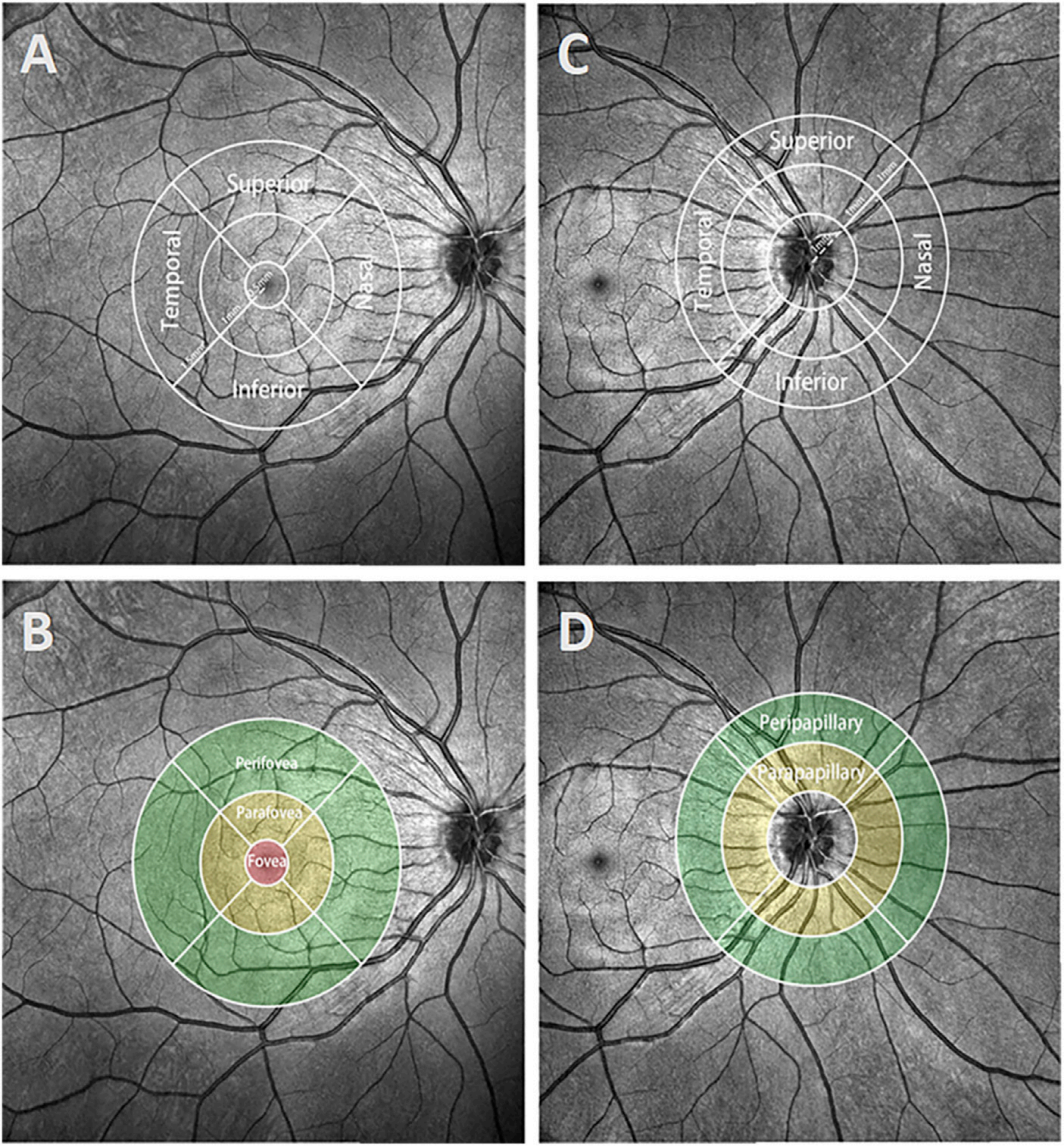
Demonstration of different regions and quadrants in the macula and optic disc. **(A and B)** The ETDRS grid of the macula was separated into regions with diameters of 1 mm (fovea), 1–3 mm (parafovea), and 3–6 mm (perifovea). The parafovea and perifovea were further divided into four quadrants: superior, inferior, nasal, and temporal. **(C, D)** The CycleTwo grid of the optic disc was separated into regions with diameters of 2 mm (papillary), 2–4 mm (parapapillary), and 4–6 mm (peripapillary). The parapapillary and peripapillary regions, like the parafovea and perifovea regions, were also separated into four quadrants.

Finally, compartmental analysis was performed (Figure 2). In the macula, the Early Treatment Diabetic Retinopathy Study (ETDRS) grid divided the region into three concentric rings with diameters of 1 mm (fovea), 1–3 mm (parafovea), and 3–6 mm (perifovea). In the optic disc, we used a CycleTwo grid and divided the regions into three rings with diameters of 2 mm (papillary), 2–4 mm (parapapillary), and 4–6 mm (peripapillary). The fovea, parafovea, and perifovea and parapapillary and peripapillary quadrants were further divided into superior (S), inferior (I), nasal (N), and temporal (T) parts.

### Reproducibility of optical coherence tomography angiography measurements

The reproducibility of the OCTA measurements was determined using intraclass correlation coefficients (ICCs) and coefficients of repeatability. Another 12 individuals’ eyes were scanned twice by an experienced observer (H.L.) and then re-scanned 3 days later by the same observer. The coefficients of repeatability were calculated to be 1.98 times the standard deviation of the changes between the two measurements and indicated that the OCTA measurements had a high degree of reproducibility.

### Statistical analysis

Categorical variables were assessed by the chi-square test and are displayed as counts (percentages). Continuous variables were assessed by ANOVA and are expressed as the mean value ±standard deviation. All values were compared using Tukey’s multiple comparisons test. Pearson’s correlation analysis was used to determine the correlation between choroidal parameters and AL and SE. A *p*-value < 0.05 was considered statistically significant. All statistical analyses in the study were performed using R (http://www.R-project.org) and Empower Stats software (www.empowerstats.com. X&Y solutions, Inc. Boston, MA).

## Results

### General characteristics

A total of 248 eye samples from 135 participants were included in this study. The demographic features of the four myopia groups are shown in Table 1. The average age of the subjects was 23.282 ± 2.223 years. 159 eye samples were obtained (65.164%) from females. A total of 125 (50.403%) eyes were right eyes. The mean SE, AL, and IOP of cohort eyes were −5.059 ± 2.849 D, 25.463 ± 1.379 mm, and 16.135 ± 2.250 mmHg, respectively. Among the four myopia groups, statistically significant differences were found in age, gender, SE, and AL (*p* < 0.05) but not in laterality or IOP (*p* > 0.05).

**TABLE 1:** Demographic features of the enrolled subjects.

Variable	Total	EM	MIM	MOM	HM	*p* Value
No. of eyes	248	43	40	65	100	—
Age, years	23.282 ± 2.223	23.953 ± 2.618	24.550 ± 2.490	22.723 ± 1.933	22.850 ± 1.822	<0.001
Female, n (%)	159 (65.164)	30 (69.767)	23 (57.500)	49 (80.328)	57 (57.000)	0.014
OD, n (%)	125 (50.403)	22 (51.163)	19 (47.500)	29 (44.615)	55 (55.000)	0.602
SE, D	−5.059 ± 2.849	−1.087 ± 1.524	−3.632 ± 1.655	−5.277 ± 2.141	−7.195 ± 1.705	<0.001
AL, mm	25.463 ± 1.379	23.196 ± 0.581	24.581 ± 0.254	25.519 ± 0.285	26.753 ± 0.542	<0.001
IOP, mmHg	16.135 ± 2.250	16.614 ± 2.600	16.150 ± 2.274	15.992 ± 2.268	16.015 ± 2.066	0.478

EM, emmetropia; MIM, mild myopia; MOM, moderate myopia; HM, high myopia; SE, spherical equivalent; AL, axial length; IOP, intraocular pressure. Data were expressed as mean ± standard deviation. Differences in values were compared by Chi-square test for categorical variables and ANOVA for continuous variables.

### Choroidal parameter analyses of the macular region

For the indicators of the macular region, the CVV, CVI, and ChT showed a marked decrease when comparing HM with EM, MIM, and MOM groups (Table 2, Supplementary Table S1). Significant differences in the global CVV were found among the EM (3.486 ± 0.992), MIM (3.238 ± 1.033), MOM (2.589 ± 1.083), and HM (1.930 ± 0.865) groups (*p* < 0.001). In the four quadrants (superior, temporal, inferior, and nasal parts) from the parafovea and perifovea regions, eyes with HM had a lower CVI than eyes with EM, MIM, and MOM (*p* < 0.001). The results showed that global CVIs in the EM, MIM, MOM, and HM groups were 0.320 ± 0.055, 0.320 ± 0.051, 0.286 ± 0.066, and 0.258 ± 0.061, respectively (*p* < 0.001). In each quadrant, the HM group had a lower CVI than the other three myopia groups (*p* < 0.001). The ChT was thinner in eyes with HM (242.753 ± 65.641) than in eyes with EM (377.532 ± 80.593), MIM (348.367 ± 78.191), or MOM (300.197 ± 87.175) (*p* < 0.001). In the superior, temporal, inferior, and nasal quadrants, ChT in the HM group was also lower than that in the other three groups (*p* < 0.001).

**TABLE 2:** Comparison of global CVV, CVI, and ChT in macular regions and papillary regions of different myopia groups.

	EM	MIM	MOM	HM	*p* Value	Post hoc
Macular						
CVV, mm³	3.486 ± 0.992	3.238 ± 1.033	2.589 ± 1.083	1.930 ± 0.865	<0.001	EM/MIM > MOM > HM
CVI	0.320 ± 0.055	0.320 ± 0.051	0.286 ± 0.066	0.258 ± 0.061	<0.001	EM/MIM > MOM > HM
ChT, μm	377.532 ± 80.593	348.367 ± 78.191	300.197 ± 87.175	242.753 ± 65.641	<0.001	EM/MIM > MOM > HM
Papillary						
CVV, mm³	1.878 ± 0.601	1.845 ± 0.689	1.514 ± 0.779	1.158 ± 0.573	<0.001	EM/MIM > MOM > HM
CVI	0.286 ± 0.044	0.295 ± 0.053	0.253 ± 0.079	0.229 ± 0.078	<0.001	EM/MIM > MOM/HM
ChT, μm	251.047 ± 55.509	236.323 ± 49.972	216.842 ± 61.050	182.536 ± 42.877	<0.001	EM/MIM > MOM > HM

EM, emmetropia; MIM, mild myopia; MOM, moderate myopia; HM, high myopia; CVV, choroidal vessel volume; CVI, choroidal vessel index; ChT, choroidal thickness. Data were expressed as mean ± standard deviation. Differences in values were compared by Chi-square test for categorical variables and analysis of variance (ANOVA) for continuous variables. All values were compared using Tukey’s multiple comparisons test.

**FIGURE 3:**
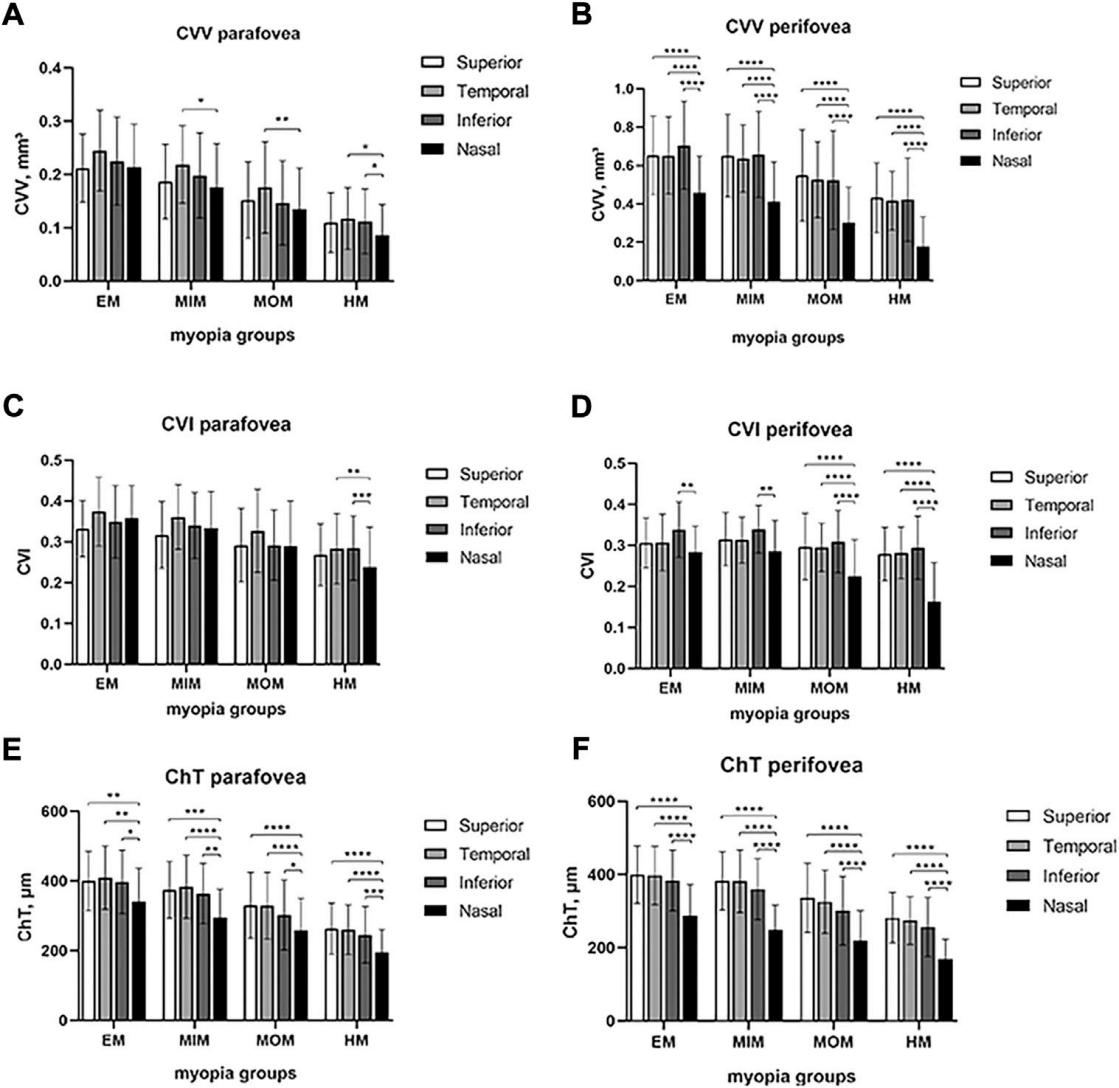
Comparison of CVV, CVI, and ChT in macular regions among four quadrants of different myopic groups. **(A)** CVV in the parafovea region. **(B)** CVV in the perifovea region. **(C)** CVI in the parafovea region. **(D)** CVI in the perifovea region. **(E)** ChT in the parafovea region. **(F)** ChT in the perifovea region. Statistical significance was marked as * (*p* < 0.05), ** (*p* < 0.01), *** (*p* < 0.001), and **** (*p* < 0.0001). EM, emmetropia; MIM, mild myopia; MOM, moderate myopia; HM, high myopia; CVV, choroidal vessel volume; CVI, choroidal vessel index; ChT, choroidal thickness.

To explore differences among superior, temporal, inferior, and nasal quadrants, comparisons were performed, as shown in Figure 3. In parafovea, the nasal CVV was slightly lower than temporal CVV in the MIM (*p* < 0.05) and MOM (*p* < 0.01) groups; also, nasal CVV was lower than temporal and inferior CVV in the HM group (*p* < 0.05). In perifovea, nasal CVV was lower than superior, temporal, and inferior CVV in each myopic group (*p* < 0.0001). In parafovea, nasal CVI was lower than temporal and inferior CVI (*p* < 0.01). In perifovea, nasal CVI was lower than inferior CVI in each myopic group (*p* < 0.01); also, nasal CVI was lower than superior and temporal CVI in the MOM and HM groups (*p* < 0.0001). In parafovea and perifovea, nasal ChT was lower than superior, temporal, and inferior ChT in each myopic group (*p* < 0.05).

### Choroidal parameter analyses of the papillary region

In the parapapillary and peripapillary regions, CVV, CVI, and ChT were lower in the HM group than in the EM, MIM, and MOM groups (Table 2, Supplementary Table S2). Global CVV was shown to be lower in the HM group (1.158 ± 0.573) than in the EM (1.878 ± 0.601), MIM (1.845 ± 0.689), and MOM (1.514 ± 0.779) groups (*p* < 0.001). In various parapapillary and peripapillary quadrants, CVV in the HM group was always the lowest compared to the EM, MIM, and MOM groups, whereas global CVI in the HM group (0.229 ± 0.078) had no significant difference from the MOM group (0.253 ± 0.079) but was lower than that in the EM (0.286 ± 0.044) and MIM (0.295 ± 0.053) groups. In the parapapillary region, superior (*p* = 0.084) and nasal (*p* = 0.070) CVI had no difference among different myopic groups. In the peripapillary region, nasal CVI had no difference among different myopic groups (*p* = 0.429). The ChT was thinner in HM eyes (182.536 ± 42.877) than in EM eyes (251.047 ± 55.509), MIM eyes (236.323 ± 49.972), and MOM (216.842 ± 61.050) eyes, globally and in every quadrant (*p* < 0.001).

**FIGURE 4:**
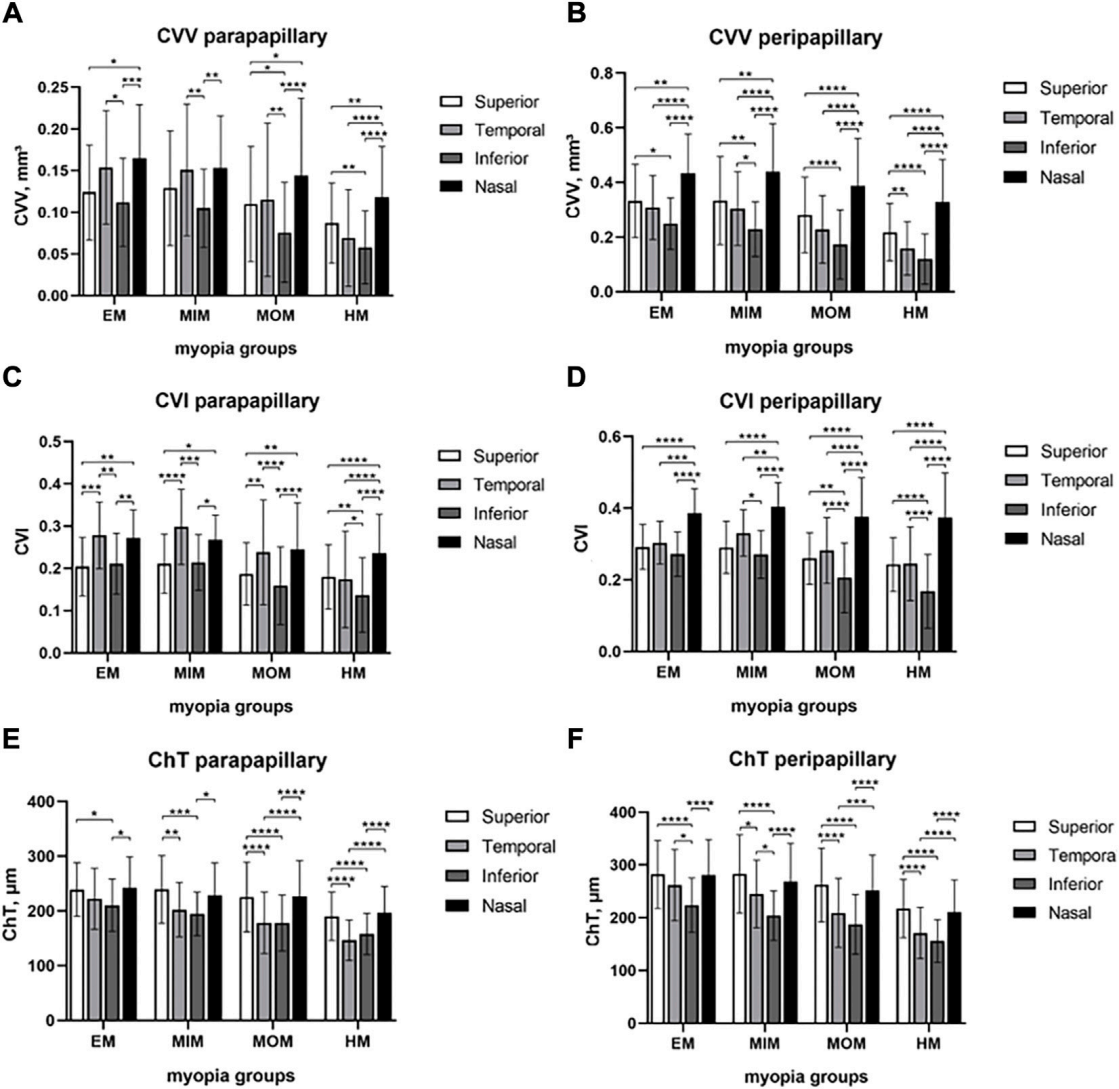
Comparison of CVV, CVI, and ChT in papillary regions among four quadrants of different myopic groups. **(A)** CVV in the parapapillary region. **(B)** CVV in the peripapillary region. **(C)** CVI in the parapapillary region. **(D)** CVI in the peripapillary region. **(E)** ChT in the parapapillary region. **(F)** ChT in the peripapillary region. Statistical significance was marked as * (*p* < 0.05), ** (*p* < 0.01), *** (*p* < 0.001), and **** (*p* < 0.0001). EM, emmetropia; MIM, mild myopia; MOM, moderate myopia; HM, high myopia; CVV, choroidal vessel volume; CVI, choroidal vessel index; ChT, choroidal thickness.

To find the difference among superior, temporal, inferior, and nasal quadrants, comparisons were performed, as shown in Figure 4. In the parapapillary region, inferior CVV was lower than temporal CVV in EM, MIM, and MOM groups (*p* < 0.05); in the HM group, inferior CVV had no difference from temporal CVV but was lower than superior and nasal CVV (*p* < 0.01). In the peripapillary region, inferior CVV was lower than superior and nasal CVV in every myopic group (*p* < 0.05); temporal CVV was lower than nasal CVV in each group (*p* < 0.0001). In the parapapillary region, inferior CVI was lower than superior and nasal CVI in each group (*p* < 0.05); inferior CVI was also lower than temporal in the HM group. In the peripapillary region, inferior CVI was lower than other quadrants’ CVI in MOM and HM groups (*p* < 0.01). In the parapapillary and peripapillary regions, ChT of inferior and temporal quadrants tended to be lower than that of other quadrants.

### Correlation between choroidal parameters with AL and SE

Pearson’s correlation analyses were used to investigate the relationship between choroidal parameters with AL and SE (Table 3). CVV (Pearson R −0.5284, *p* < 0.0001), CVI (Pearson R −0.3667, *p* < 0.0001), and ChT (Pearson R −0.5840, *p* < 0.0001) were negatively correlated with AL. CVV (Pearson R 0.4995, *p* < 0.0001), CVI (Pearson R 0.3653, *p* < 0.0001), and ChT (Pearson R 0.5401, *p* < 0.0001) were negatively correlated with SE.

**TABLE 3:** Correlation of choroidal parameters with AL and SE.

Parameter	AL		SE	
	Pearson R	*p* Value	Pearson R	*p* Value
CVV	−0.5284	<0.0001	0.4995	<0.0001
CVI	−0.3667	<0.0001	0.3653	<0.0001
Vessel density of CC	−0.3205	<0.0001	0.3929	<0.0001
ChT	−0.5840	<0.0001	0.5401	<0.0001

CVV, choroidal vessel volume; CVI, choroidal vessel index; CC, choriocapillaris; ChT, choroidal thickness. β, R, and *p* values were derived using Pearson’s correlation analysis.

## Discussion

SS-OCTA has gained increasing prevalence in recent years. Its high resolution, rapid scanning rate, and noninvasive features enable research on multiple fundus diseases (Li et al., 2022; Terada et al., 2022; Vagge et al., 2022). In our study, two wide scanning areas of 6 mm × 6 mm were used, covering the macula and the optic disc.

3D CVV and CVI analyses are the highlights of our study. Previously, CVI was used to quantify choroid blood perfusion in central serous chorioretinopathy (Yang et al., 2020), Stargardt disease (Ratra et al., 2018), and retinitis pigmentosa (Shen et al., 2020). Notably, the majority of these investigations applied a binarization algorithm to calculate the CVI. They analyzed images captured with enhanced depth imaging optical coherence tomography (EDI-OCT) or SD-OCTA. Only horizontal and vertical B-scans crossing through the fovea were assessed, which produced two-dimensional (2D) data (Ratra et al., 2018; Shen et al., 2020; Yang et al., 2020; Wu et al., 2021). The result of 2D CVI was relatively limited and lost the volumetric feature. Thus, 3D CVV and CVI data applied in our study are more powerful and meaningful for illustrating choroidal blood perfusion alterations associated with myopia.

One major finding of our study was that CVV, CVI, and ChT were lower in eyes with longer AL or eyes with higher SE, which indicated that large and medium vessels might start to change in myopic eyes even without maculopathy. Our result was in line with those of previous studies. A negative correlation between AL and ChT was reported not only in young myopic adults but also in experimental animals (Hirata and Negi, 1998; Mastropasqua et al., 2019). Furthermore, Mastropasqua et al. (2019) revealed that when myopia progressed, choriocapillaris (CC) blood perfusion decreased in both the macular and optic disc areas. Similar findings were also reported by Panda-Jonas et al. (2021) and Su et al. (2020). Very recently, Xu et al. (2021) found that 3D CVI and blood perfusion of CC decreased with increasing severity of myopia.

The plausible explanation for these changes might be pathological damage caused by myopia. Studies have shown that in myopic eyes, scleral hypoxia could induce chorioretinal perfusion reduction (Wu et al., 2018). Also, stretching and thinning of the retina and choroid might result in vascular compromise and reduce vascular perfusion (Khan et al., 2020). Our results indicated that the greater the degree of myopia, the thinner the ChT, which was probably due to loss of choroidal vascular components. The decrease in 3D CVV illustrated that choroidal medium and large vessels gradually compromise as the eye is overly elongated. The decrease in 3D CVI, defined as the ratio of choroidal lumina and choroidal total volume (lumina plus stroma), implied that loss of the choroidal vascular component might be more severe than loss of stroma during myopia development.

Intriguingly, Yazdani et al. (2021) found a larger CVI in the myopic group than in the emmetropic group, which is converse to our result. They ascribed the reduction in choroidal thickness mostly to choroidal stromal thinning rather than vascular thinning. The reason might be associated with different algorithms used. As we discussed earlier, 2D CVI only included two B-scan pictures centered on the fovea. In comparison, our research analyzed 3D CVI based on 1,024 × 1024 B-scans and included a larger sample size, covered a larger region, and spanned a broader AL.

The other major finding was that the average ChT, CVV, and CVI decreased more dramatically on the nasal side of the macula and the temporal and inferior sides of the optic disc. To our knowledge, compartmental analysis was rarely used in previous research, especially in the choroid. The underlying mechanisms might be associated with weakness of the papillo-macular bundle. As the degree of myopia became severe, parapapillary gamma and delta zones enlarged and choroidal perfusion gradually decreased (Jonas et al., 2012; Kim et al., 2020). During this process, the symbolized area of change was the papillo-macular bundle.

The results of our study were based on a young myopic population (range: 18–30 years), and further studies are needed to investigate the changes in other adult age groups. Lu et al. (2022) showed a negative correlation between ChT and AL in a myopic group with a mean age of 66 years (range: 40–80 years) and the highest negative correlation between ChT and AL on the nasal side, which is consistent with our results. As for the controversial results of choroidal vascularity and CVI, Yazdani et al. (2021) found that 2D CVI was larger in the myopic group than in the emmetropic group (range: 20–38 years), which is contrary to our result. However, Wu et al. (2021) and Xu et al. (2021) showed a negative correlation between CVI and AL. The former study indicated that topographic changes in 2D CVI were prominent in the temporal and inferior macular regions, with an age range of 20–27 years. The latter study demonstrated that changes in 3D CVI were larger in the nasal in high- than low-myopia groups with an age range of 19–62 years. For the inconsistency of these findings and the differences in the decrease of stromal and vascular components in each region, we are subsequently collecting other age groups of myopic patients to validate and compensate for the limitations of this study. (Page Line).

However, this study also has limitations. Only young subjects were included in our study. Additionally, age and gender differences were found among the four myopic groups. Although the difference in age among the four groups was only 1.827 years and might influence the choroidal parameters to a very small degree, it would be better if we enlarged the sample size.

## Conclusion

In this study, we found a strong association between various choroidal parameters and AL. Lower CVV, CVI, and ChT values were found in eyes with a greater AL in young myopic adults. The decrease of the CVV and CVI was much faster in the region between the macula and optic disc in myopic eyes. These findings demonstrated the 3D choroidal change in myopic development and highlighted that CVV and CVI alterations in the papillo-macular bundle were important indicators of myopia.

## Data Availability

The original contributions presented in the study are included in the article/Supplementary Material; further inquiries can be directed to the corresponding authors.

## References

[B1] Agrawal R. Ding J. Sen P. Rousselot A. Chan A. Nivison-Smith L. (2020). Exploring choroidal angioarchitecture in health and disease using choroidal vascularity index. Prog. Retin. Eye Res. 77, 100829. 10.1016/j.preteyeres.2020.100829 31927136

[B2] Al-Sheikh M. Phasukkijwatana N. Dolz-Marco R. Rahimi M. Iafe N. A. Freund K. B. (2017). Quantitative OCT angiography of the retinal microvasculature and the choriocapillaris in myopic eyes. Invest. Ophthalmol. Vis. Sci. 58, 2063–2069. 10.1167/iovs.16-21289 28388703

[B3] Baird P. N. Saw S. M. Lanca C. Guggenheim J. A. Smith E. L. Iii Zhou X. (2020). Myopia. Nat. Rev. Dis. Prim. 6, 99. 10.1038/s41572-020-00231-4 33328468

[B4] Bennett A. G. Rudnicka A. R. Edgar D. F. (1994). Improvements on Littmann's method of determining the size of retinal features by fundus photography. Graefes. Arch. Clin. Exp. Ophthalmol. 232, 361–367. 10.1007/BF00175988 8082844

[B5] Chiang S. T. Phillips J. R. Backhouse S. (2015). Effect of retinal image defocus on the thickness of the human choroid. Ophthalmic Physiol. Opt. 35, 405–413. 10.1111/opo.12218 26010292

[B6] Corbelli E. Sacconi R. Battista M. Bacherini D. Miere A. Borrelli E. (2022). Choroidal vascularity index in eyes with central macular atrophy secondary to age-related macular degeneration and Stargardt disease. Graefes. Arch. Clin. Exp. Ophthalmol. 260, 1525–1534. 10.1007/s00417-021-05337-310.1007/s00417-021-05337-3 35048199

[B7] Hirata A. Negi A. (1998). Morphological changes of choriocapillaris in experimentally induced chick myopia. Graefes. Arch. Clin. Exp. Ophthalmol. 236, 132–137. 10.1007/s004170050053 9498124

[B8] Holden B. A. Fricke T. R. Wilson D. A. Jong M. Naidoo K. S. Sankaridurg P. (2016). Global prevalence of myopia and high myopia and temporal trends from 2000 through 2050. Ophthalmology 123, 1036–1042. 10.1016/j.ophtha.2016.01.006 26875007

[B9] Hwang B. E. Kwak J. H. Kim J. Y. Kim R. Y. Kim M. Park Y. G. (2022). Quantitative analysis of choroidal blood flow parameters in optical coherence tomography and angiography in central serous chorioretinopathy. Graefes. Arch. Clin. Exp. Ophthalmol. 260, 2111–2120. 10.1007/s00417-022-05588-8 35201403

[B10] Jonas J. B. Jonas S. B. Jonas R. A. Holbach L. Dai Y. Sun X. (2012). Parapapillary atrophy: Histological gamma zone and delta zone. PLoS One 7, e47237. 10.1371/journal.pone.0047237 23094040 PMC3475708

[B11] Kashani A. H. Chen C. L. Gahm J. K. Zheng F. Richter G. M. Rosenfeld P. J. (2017). Optical coherence tomography angiography: A comprehensive review of current methods and clinical applications. Prog. Retin. Eye Res. 60, 66–100. 10.1016/j.preteyeres.2017.07.002 28760677 PMC5600872

[B12] Khan M. H. Lam A. Armitage J. A. Hanna L. To C. H. Gentle A. (2020). Impact of axial eye size on retinal microvasculature density in the macular region. J. Clin. Med. 9, 2539. 10.3390/jcm9082539 PMC7463769 32781548

[B13] Kim G. N. Lee E. J. Kim T. W. (2020). Microstructure of nonjuxtapapillary microvasculature dropout in healthy myopic eyes. Invest. Ophthalmol. Vis. Sci. 61, 36. 10.1167/iovs.61.2.36 PMC7329630 32084265

[B14] Lee S. W. Yu S. Y. Seo K. H. Kim E. S. Kwak H. W. (2014). Diurnal variation in choroidal thickness in relation to sex, axial length, and baseline choroidal thickness in healthy Korean subjects. Retina 34, 385–393. 10.1097/IAE.0b013e3182993f29 23873165

[B15] Li J. Zhou H. Feinstein M. Wong J. Wang R. K. Chan L. (2022). Choriocapillaris changes in myopic macular degeneration. Transl. Vis. Sci. Technol. 11, 37. 10.1167/tvst.11.2.37 PMC8883151 35201337

[B16] Li M. Jin E. Dong C. Zhang C. Zhao M. Qu J. (2018). The repeatability of superficial retinal vessel density measurements in eyes with long axial length using optical coherence tomography angiography. BMC Ophthalmol. 18, 326. 10.1186/s12886-018-0992-y 30558579 PMC6297956

[B17] Li M. Yang Y. Jiang H. Gregori G. Roisman L. Zheng F. (2017). Retinal microvascular network and microcirculation assessments in high myopia. Am. J. Ophthalmol. 174, 56–67. 10.1016/j.ajo.2016.10.018 27818204 PMC5253241

[B18] Liu M. Wang P. Hu X. Zhu C. Yuan Y. Ke B. (2021). Myopia-related stepwise and quadrant retinal microvascular alteration and its correlation with axial length. Eye 35, 2196–2205. 10.1038/s41433-020-01225-y 33087885 PMC8302696

[B19] Lu H. C. Chen H. Y. Huang C. J. Chu P. H. Wu L. S. Tsai C. Y. (2022). Predicting axial length from choroidal thickness on optical coherence tomography images with machine learning based algorithms. Front. Med. 9, 850284. 10.3389/fmed.2022.850284 PMC9273745 35836947

[B20] Mastropasqua R. Viggiano P. Borrelli E. Evangelista F. Libertini D. Di Antonio L. (2019). *In vivo* mapping of the choriocapillaris in high myopia: A widefield swept source optical coherence tomography angiography. Sci. Rep. 9, 18932. 10.1038/s41598-019-55192-w 31831754 PMC6908654

[B21] Moghimi S. Hosseini H. Riddle J. Lee G. Y. Bitrian E. Giaconi J. (2012). Measurement of optic disc size and rim area with spectral-domain OCT and scanning laser ophthalmoscopy. Invest. Ophthalmol. Vis. Sci. 53, 4519–4530. 10.1167/iovs.11-8362 22577077

[B22] Morgan I. G. French A. N. Ashby R. S. Guo X. Ding X. He M. (2018). The epidemics of myopia: Aetiology and prevention. Prog. Retin. Eye Res. 62, 134–149. 10.1016/j.preteyeres.2017.09.004 28951126

[B23] Morgan I. G. Ohno-Matsui K. Saw S. M. (2012). Myopia. Lancet 379, 1739–1748. 10.1016/S0140-6736(12)60272-4 22559900

[B24] Nicolini N. Tombolini B. Barresi C. Pignatelli F. Lattanzio R. Bandello F. (2022). Assessment of diabetic choroidopathy using ultra-widefield optical coherence tomography. Transl. Vis. Sci. Technol. 11, 35. 10.1167/tvst.11.3.35 PMC8976931 35353150

[B25] Panda-Jonas S. Holbach L. Jonas J. B. (2021). Choriocapillaris thickness and density in axially elongated eyes. Acta Ophthalmol. 99, 104–110. 10.1111/aos.14486 32562378

[B26] Ratra D. Tan R. Jaishankar D. Khandelwal N. Gupta A. Chhablani J. (2018). Choroidal structural changes and vascularity index in Stargardt disease on swept source optical coherence tomography. Retina 38, 2395–2400. 10.1097/IAE.0000000000001879 29016459

[B27] Ruiz-Medrano J. Montero J. A. Flores-Moreno I. Arias L. García-Layana A. Ruiz-Moreno J. M. (2019). Myopic maculopathy: Current status and proposal for a new classification and grading system (ATN). Prog. Retin. Eye Res. 69, 80–115. 10.1016/j.preteyeres.2018.10.005 30391362

[B28] Shen C. Li Y. Wang Q. Chen Y. N. Li W. Wei W. B. (2020). Choroidal vascular changes in retinitis pigmentosa patients detected by optical coherence tomography angiography. BMC Ophthalmol. 20, 384. 10.1186/s12886-020-01640-5 32993583 PMC7523071

[B29] Siggel R. Spital C. Lentzsch A. Liakopoulos S. (2022). Optical coherence tomography angiography for the detection of macular neovascularization-comparison of en face versus cross-sectional view. Eye (Lond). 10.1038/s41433-021-01892-5 PMC9873677 34992250

[B30] Singh S. R. Vupparaboina K. K. Goud A. Dansingani K. K. Chhablani J. (2019). Choroidal imaging biomarkers. Surv. Ophthalmol. 64, 312–333. 10.1016/j.survophthal.2018.11.002 30496750

[B31] Su L. Ji Y. S. Tong N. Sarraf D. He X. Sun X. (2020). Quantitative assessment of the retinal microvasculature and choriocapillaris in myopic patients using swept-source optical coherence tomography angiography. Graefes. Arch. Clin. Exp. Ophthalmol. 258, 1173–1180. 10.1007/s00417-020-04639-2 32144487

[B32] Sun G. Chen C. Jiang J. Yi Z. Wang X. Miao Q. (2021). New insights into the association between choroidal vessels and choriocapillaris in normal eyes. Retina 41, 2612–2619. 10.1097/IAE.0000000000003238 34173364

[B33] Terada N. Murakami T. Uji A. Ishihara K. Dodo Y. Nishikawa K. (2022). The intercapillary space spectrum as a marker of diabetic retinopathy severity on optical coherence tomography angiography. Sci. Rep. 12, 3089. 10.1038/s41598-022-07128-0 35197526 PMC8866469

[B34] Vagge A. Nucci P. Ferro Desideri L. Testa V. Scaramuzzi M. Siccardi G. (2022). Evaluation of macular vessel density changes after strabismus surgery using optical coherence tomography angiography. J. AAPOS 26 (2), 71.e1–71.e4. 10.1016/j.jaapos.2021.11.011 35307544

[B35] Wang W. Guo X. Chen Y. Xiong K. Gong X. Yuan M. (2022). Choriocapillaris perfusion assessed using swept source optical coherence tomographic angiography and the severity of diabetic retinopathy. Br. J. Ophthalmol. 2021, 320163. 10.1136/bjophthalmol-2021-320163 35115302

[B36] Woodman-Pieterse E. C. Read S. A. Collins M. J. Alonso-Caneiro D. (2015). Regional changes in choroidal thickness associated with accommodation. Invest. Ophthalmol. Vis. Sci. 56, 6414–6422. 10.1167/iovs.15-17102 26444722

[B37] Wu H. Chen W. Zhao F. Zhou Q. Reinach P. S. Deng L. (2018). Scleral hypoxia is a target for myopia control. Proc. Natl. Acad. Sci. U. S. A. 115, E7091–E7100. 10.1073/pnas.1721443115 29987045 PMC6064999

[B38] Wu H. Zhang G. Shen M. Xu R. Wang P. Guan Z. (2021). Assessment of choroidal vascularity and choriocapillaris blood perfusion in anisomyopic adults by SS-oct/OCTA. Invest. Ophthalmol. Vis. Sci. 62, 8. 10.1167/iovs.62.1.8 PMC7797932 33393974

[B39] Xu A. Sun G. Duan C. Chen Z. Chen C. (2021). Quantitative assessment of three-dimensional choroidal vascularity and choriocapillaris flow signal voids in myopic patients using SS-OCTA. Diagnostics 11 (11), 1948. 10.3390/diagnostics11111948 34829297 PMC8618547

[B40] Yang J. Wang E. Yuan M. Chen Y. (2020). Three-dimensional choroidal vascularity index in acute central serous chorioretinopathy using swept-source optical coherence tomography. Graefes. Arch. Clin. Exp. Ophthalmol. 258, 241–247. 10.1007/s00417-019-04524-7 31724090

[B41] Yang S. Zhou M. Lu B. Zhang P. Zhao J. Kang M. (2017). Quantification of macular vascular density using optical coherence tomography angiography and its relationship with retinal thickness in myopic eyes of young adults. J. Ophthalmol. 2017, 1397179. 10.1155/2017/1397179 29318037 PMC5727759

[B42] Yang Y. Wang J. Jiang H. Yang X. Feng L. Hu L. (2016). Retinal microvasculature alteration in high myopia. Invest. Ophthalmol. Vis. Sci. 57, 6020–6030. 10.1167/iovs.16-19542 27820633

[B43] Yazdani N. Ehsaei A. Hoseini-Yazdi H. Shoeibi N. Alonso-Caneiro D. Collins M. J. (2021). Wide-field choroidal thickness and vascularity index in myopes and emmetropes. Ophthalmic Physiol. Opt. 41, 1308–1319. 10.1111/opo.12875 34487376

