# Supplementary material for: Compartmental analysis of three-dimensional choroidal vascularity and thickness of myopic eyes in young adults using SS-OCTA
Source: Front Physiol. 2022 Sep 7;13:916323. doi: 10.3389/fphys.2022.916323 (PMC9490056; doi:10.3389/fphys.2022.916323)
Supplement: Supplementary file 1 [file Table1.DOCX]

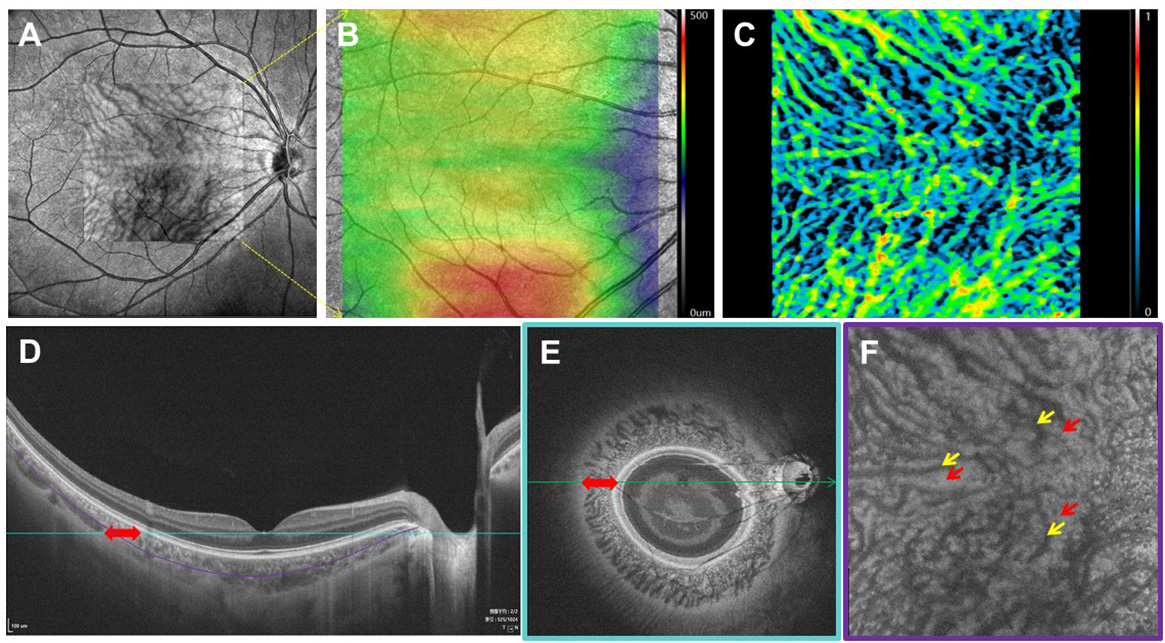


**Supplementary Figure 1.** Illustration of the choroidal morphology and the choroidal parameters from various dimensions by SS-OCTA.

(**A**) A raw en-face SS-OCTA image in the macular region of 6 mm × 6 mm. (**B**) A 6 mm × 6 mm map of the macular choroidal thickness (ChT). (**C**) A 6 mm × 6 mm map of the macular three-dimensional (3D) choroidal vessel index (CVI). (**D**) A B-scan image of one patient. (**E**) An en-face image corresponds to the blue line in **D**. The red bidirectional arrows in **D** and **E** represent the choroid. (**F**) An en-face image corresponds to the purple curve in **D**. The yellow arrows represent the choroidal lumen and the red arrows represent the choroidal stroma.

**Supplementary** **Table 1.** Comparison of CVV, CVI and ChT in macular regions among four quadrants of different myopic groups.

|  | **EM** | **MIM** | **MOM** | **HM** | **P value** | **Post hoc** |
| --- | --- | --- | --- | --- | --- | --- |
| **CVV, mm³** |  |  |  |  |  |  |
| Parafovea |  |  |  |  |  |  |
| Superior | 0.211 ± 0.064 | 0.186 ± 0.070 | 0.152 ± 0.072 | 0.110 ± 0.056 | <0.001 | EM>MIM/MOM>HM |
| Temporal | 0.245 ± 0.077 | 0.218 ± 0.074 | 0.176 ± 0.086 | 0.117 ± 0.058 | <0.001 | EM/MIM>MOM>HM |
| Inferior | 0.223 ± 0.083 | 0.197 ± 0.080 | 0.147 ± 0.079 | 0.112 ± 0.061 | <0.001 | EM/MIM>MOM>HM |
| Nasal | 0.213 ± 0.081 | 0.175 ± 0.083 | 0.135 ± 0.077 | 0.086 ± 0.058 | <0.001 | EM/MIM>MOM>HM |
| Perifovea |  |  |  |  |  |  |
| Superior | 0.650 ± 0.204 | 0.650 ± 0.217 | 0.548 ± 0.238 | 0.432 ± 0.182 | <0.001 | EM/MIM>MOM>HM |
| Temporal | 0.652 ± 0.203 | 0.635 ± 0.177 | 0.526 ± 0.198 | 0.417 ± 0.153 | <0.001 | EM/MIM>MOM>HM |
| Inferior | 0.703 ± 0.232 | 0.661 ± 0.226 | 0.524 ± 0.257 | 0.422 ± 0.217 | <0.001 | EM/MIM>MOM>HM |
| Nasal | 0.455 ± 0.192 | 0.414 ± 0.210 | 0.302 ± 0.185 | 0.180 ± 0.154 | <0.001 | EM/MIM>MOM>HM |
| **CVI** |  |  |  |  |  |  |
| Parafovea |  |  |  |  |  |  |
| Superior | 0.330 ± 0.069 | 0.315 ± 0.082 | 0.292 ± 0.090 | 0.268 ± 0.076 | <0.001 | EM>MIM>MOM/HM |
| Temporal | 0.374 ± 0.085 | 0.361 ± 0.080 | 0.327 ± 0.102 | 0.283 ± 0.086 | <0.001 | EM>MIM/MOM>HM |
| Inferior | 0.347 ± 0.089 | 0.339 ± 0.082 | 0.292 ± 0.086 | 0.285 ± 0.079 | <0.001 | EM/MIM>MOM/HM |
| Nasal | 0.356 ± 0.079 | 0.332 ± 0.089 | 0.289 ± 0.111 | 0.238 ± 0.098 | <0.001 | EM/MIM/MOM>HM |
| Perifovea |  |  |  |  |  |  |
| Superior | 0.305 ± 0.061 | 0.315 ± 0.066 | 0.297 ± 0.081 | 0.279 ± 0.065 | 0.025 | MIM>HM |
| Temporal | 0.307 ± 0.070 | 0.313 ± 0.057 | 0.295 ± 0.059 | 0.282 ± 0.063 | 0.030 | MIM>HM |
| Inferior | 0.338 ± 0.068 | 0.341 ± 0.058 | 0.309 ± 0.076 | 0.294 ± 0.077 | <0.001 | EM/MIM>HM |
| Nasal | 0.282 ± 0.062 | 0.287 ± 0.076 | 0.225 ± 0.089 | 0.163 ± 0.095 | <0.001 | EM/MIM>MOM>HM |
| **ChT, μm** |  |  |  |  |  |  |
| Parafovea |  |  |  |  |  |  |
| Superior | 400.287 ± 85.028 | 374.367 ± 81.770 | 330.270 ± 94.668 | 263.270 ± 73.716 | <0.001 | EM/MIM>MOM>HM |
| Temporal | 409.628 ± 90.875 | 383.561 ± 90.976 | 329.285 ± 95.596 | 260.443 ± 71.229 | <0.001 | EM/MIM>MOM>HM |
| Inferior | 397.362 ± 90.861 | 363.853 ± 86.825 | 302.772 ± 100.313 | 245.716 ± 80.916 | <0.001 | EM/MIM>MOM>HM |
| Nasal | 341.273 ± 95.282 | 295.699 ± 81.111 | 258.361 ± 91.711 | 195.779 ± 64.793 | <0.001 | EM/MIM/MOM>HM; EM>MOM |
| Perifovea |  |  |  |  |  |  |
| Superior | 399.554 ± 78.851 | 382.501 ± 79.622 | 336.431 ± 94.794 | 281.822 ± 68.755 | <0.001 | EM/MIM>MOM>HM |
| Temporal | 397.096 ± 79.916 | 381.380 ± 85.206 | 325.613 ± 86.213 | 274.300 ± 65.407 | <0.001 | EM/MIM>MOM>HM |
| Inferior | 383.566 ± 82.859 | 359.779 ± 83.757 | 301.179 ± 93.269 | 256.382 ± 80.633 | <0.001 | EM/MIM>MOM>HM |
| Nasal | 287.046 ± 85.539 | 248.671 ± 68.265 | 219.762 ± 81.624 | 169.522 ± 53.744 | <0.001 | EM/MIM/MOM>HM; EM>MOM |

EM, emmetropia; MIM, mild myopia; MOM, moderate myopia; HM, high myopia; CVV, choroidal vessel volume; CVI, choroidal vessel index; ChT, choroidal thickness.

Data were expressed as mean ± standard deviation. Differences of values were compared by Chi-square test for categorical variables and analysis of variance (ANOVA) for continuous variables. All values were compared using Tukey’s multiple comparisons test.

**Supplementary Table 2.** Comparison of CVV, CVI and ChT in papillary regions among four quadrants of different myopic groups.

|  | **EM** | **MIM** | **MOM** | **HM** | **P value** | **Post hoc** |
| --- | --- | --- | --- | --- | --- | --- |
| **CVV, mm³** |  |  |  |  |  |  |
| Parapapillary |  |  |  |  |  |  |
| Superior | 0.124 ± 0.057 | 0.129 ± 0.069 | 0.110 ± 0.069 | 0.087 ± 0.048 | <0.001 | EM/MIM>HM |
| Temporal | 0.154 ± 0.068 | 0.151 ± 0.079 | 0.115 ± 0.092 | 0.069 ± 0.058 | <0.001 | EM/MIM>MOM>HM |
| Inferior | 0.112 ± 0.053 | 0.105 ± 0.047 | 0.076 ± 0.060 | 0.058 ± 0.044 | <0.001 | EM/MIM>MOM/HM |
| Nasal | 0.165 ± 0.064 | 0.153 ± 0.063 | 0.144 ± 0.093 | 0.118 ± 0.061 | 0.001 | EM/MIM>HM |
| Peripapillary |  |  |  |  |  |  |
| Superior | 0.332 ± 0.134 | 0.334 ± 0.161 | 0.281 ± 0.138 | 0.218 ± 0.105 | <0.001 | EM/MIM/MOM>HM |
| Temporal | 0.308 ± 0.117 | 0.304 ± 0.135 | 0.228 ± 0.124 | 0.159 ± 0.098 | <0.001 | EM/MIM>MOM>HM |
| Inferior | 0.249 ± 0.094 | 0.229 ± 0.100 | 0.173 ± 0.127 | 0.120 ± 0.092 | <0.001 | EM/MIM>HM |
| Nasal | 0.434 ± 0.143 | 0.439 ± 0.176 | 0.388 ± 0.173 | 0.328 ± 0.156 | <0.001 | EM/MIM/MOM>HM |
| **CVI** |  |  |  |  |  |  |
| Parapapillary |  |  |  |  |  |  |
| Superior | 0.204 ± 0.069 | 0.211 ± 0.070 | 0.187 ± 0.074 | 0.180 ± 0.076 | 0.084 | - |
| Temporal | 0.278 ± 0.079 | 0.298 ± 0.089 | 0.238 ± 0.124 | 0.174 ± 0.114 | <0.001 | EM/MIM>MOM>HM |
| Inferior | 0.211 ± 0.072 | 0.214 ± 0.066 | 0.159 ± 0.092 | 0.137 ± 0.088 | <0.001 | EM/MIM>MOM/HM |
| Nasal | 0.272 ± 0.066 | 0.268 ± 0.058 | 0.245 ± 0.110 | 0.236 ± 0.092 | 0.070 | - |
| Peripapillary |  |  |  |  |  |  |
| Superior | 0.292 ± 0.063 | 0.291 ± 0.073 | 0.260 ± 0.072 | 0.243 ± 0.075 | 0.025 | EM/MIM>HM |
| Temporal | 0.304 ± 0.060 | 0.331 ± 0.065 | 0.282 ± 0.092 | 0.245 ± 0.103 | 0.030 | EM/MIM>MOM>HM |
| Inferior | 0.272 ± 0.062 | 0.271 ± 0.067 | 0.206 ± 0.097 | 0.168 ± 0.103 | <0.001 | EM/MIM>MOM>HM |
| Nasal | 0.387 ± 0.067 | 0.405 ± 0.066 | 0.377 ± 0.108 | 0.375 ± 0.123 | 0.429 | - |
| **ChT, μm** |  |  |  |  |  |  |
| Parapapillary |  |  |  |  |  |  |
| Superior | 239.246 ± 48.773 | 239.347 ± 61.782 | 225.436 ± 63.594 | 190.326 ± 44.018 | <0.001 | EM/MIM/MOM>HM |
| Temporal | 222.389 ± 55.851 | 202.289 ± 49.806 | 178.318 ± 56.113 | 146.638 ± 36.976 | <0.001 | EM/MIM/MOM>HM; EM>MOM |
| Inferior | 210.441 ± 47.639 | 194.636 ± 39.714 | 177.952 ± 51.080 | 157.972 ± 37.948 | <0.001 | EM/MIM>MOM/HM; EM>MOM |
| Nasal | 242.605 ± 56.369 | 229.112 ± 58.878 | 226.891 ± 64.954 | 196.677 ± 47.995 | <0.001 | EM/MIM/MOM>HM |
| Peripapillary |  |  |  |  |  |  |
| Superior | 282.152 ± 64.257 | 283.220 ± 74.303 | 262.084 ± 69.903 | 217.412 ± 55.229 | <0.001 | EM/MIM/MOM>HM |
| Temporal | 261.998 ± 67.648 | 245.067 ± 63.994 | 209.175 ± 65.419 | 171.224 ± 48.369 | <0.001 | EM/MIM>MOM>HM |
| Inferior | 223.765 ± 51.363 | 204.092 ± 46.806 | 187.614 ± 56.553 | 156.005 ± 40.526 | <0.001 | EM/MIM/MOM>HM; EM>MOM |
| Nasal | 281.029 ± 67.246 | 268.248 ± 73.043 | 251.492 ± 66.960 | 211.105 ± 60.249 | <0.001 | EM/MIM/MOM>HM |

EM, emmetropia; MIM, mild myopia; MOM, moderate myopia; HM, high myopia; CVV, choroidal vessel volume; CVI, choroidal vessel index; ChT, choroidal thickness.

Data were expressed as mean ± standard deviation. Differences of values were compared by Chi-square test for categorical variables and analysis of variance (ANOVA) for continuous variables. All values were compared using Tukey’s multiple comparisons test.
